# Evaluation of the association between electrocardiogram parameters and left cardiac remodeling in dogs with myxomatous mitral valve disease

**DOI:** 10.14202/vetworld.2022.2072-2083

**Published:** 2022-08-27

**Authors:** Mizuki Ogawa, Haruka Ogi, Hirosumi Miyakawa, Huai-Hsun Hsu, Yuichi Miyagawa, Naoyuki Takemura

**Affiliations:** Laboratory of Veterinary Internal Medicine II, School of Veterinary Medicine, Nippon Veterinary and Life Science University, Tokyo, Japan

**Keywords:** left atrial enlargement, left ventricular enlargement, PR interval, rate of change, R-wave amplitude

## Abstract

**Background and Aim:**

Electrocardiography (ECG) is an adjunct for cardiac enlargement diagnosis. However, its efficacy in assessing left cardiac remodeling (left atrial and left ventricular enlargement) in dogs with myxomatous mitral valve disease (MMVD) remains unclear. This study aimed to evaluate the association between ECG parameters and left cardiac remodeling and to investigate whether the rate of change in ECG waveforms in the same individual reflected left cardiac remodeling in dogs with MMVD.

**Materials and Methods:**

This retrospective study included 20 healthy dogs and 140 dogs with MMVD. Data on clinical variables were obtained through physical examination, thoracic radiography, and echocardiography. The ECG parameters were the P-wave duration, PR interval, QRS complex duration, P-wave amplitude, R-wave amplitude, and mean electrical axis. Dogs with examination data that could be obtained multiple times during the study period were classified into the non-progressive and progressive groups.

**Results:**

Only the P-wave and QRS complex durations were selected as significant variables associated with imaging test parameters (p < 0.05); they had a relatively higher discriminatory ability for the left cardiac remodeling than other ECG parameters. The rates of change in the PR interval and R-wave amplitude were significantly higher in the progressive group than in the non-progressive group.

**Conclusion:**

In dogs with MMVD, the P-wave and QRS complex durations were significantly correlated with the left cardiac remodeling indicators. Furthermore, an increased rate of change in the PR interval and R-wave amplitude in the same individual may indicate left cardiac remodeling.

## Introduction

Myxomatous mitral valve disease (MMVD) is the most common type of heart disease in dogs [1, 2]. As MMVD progresses irreversibly, clinical stage progression should be monitored regularly. Based on the consensus statements established by the American College of Veterinary Internal Medicine (ACVIM) for MMVD, the condition is classified into stages A, B1, B2, C, and D according to the degree of progression [3]. Asymptomatic dogs with MMVD are classified as ACVIM stage B1 or B2 according to the absence or presence of the left cardiac remodeling (left atrial [LA] and left ventricular [LV] enlargement). In addition, it is recommended that dogs with stage B2 disease be treated with drugs such as pimobendan before the onset of clinical signs [4]. Imaging tests, including thoracic radiography and echocardiography, are mainly used to identify left cardiac remodeling. In humans, electrocardiography (ECG) is an ancillary test for detecting left cardiac enlargement [5, 6]. In dogs, changes in the shapes of ECG waveforms are considered to indicate left cardiac enlargement like in humans, and each ECG waveform has a reference value for the left heart enlargement [7, 8]. However, it has not been determined whether each ECG waveform accurately reflects left heart remodeling in dogs with MMVD, as these reference values are based on studies of certain breeds and diseases [9].

Several studies have compared the association between each ECG waveform and the left cardiac enlargement findings on imaging tests; however, these studies compared each ECG waveform with the vertebral heart size (VHS) and the left atrium/aortic ratio (LA/Ao) [10, 11]. The new MMVD guidelines recently published by the ACVIM that evaluate cardiac enlargement in dogs with MMVD included vertebral left atrial size (VLAS) and LV internal diameter in diastole normalized for body weight (LVIDDN) in addition to the traditional diagnostic indicators of the left cardiac enlargement, VHS, and LA/Ao [3]. Therefore, evaluation of the utility of ECG to estimate left cardiac enlargement in dogs with MMVD should include VLAS and LVIDDN. Moreover, as these imaging test parameters come from only one cross-sectional imaging finding, they alone cannot wholly reflect left cardiac enlargement. Therefore, to accurately investigate the association between the ECG waveform and left cardiac remodeling, the diagnosis of left cardiac remodeling should be based on a combination of several imaging test parameters. The ECG waveforms in dogs exhibit inter-individual variability based on differences in body weight and chest size [8, 12, 13]. The thoracic morphology of dogs varies significantly according to the breed. Therefore, conventional ECG waveform reference values may over- or underestimate the left heart enlargement owing to the influence of body weight and chest circumference [7]. Therefore, evaluating the intra-individual variability of each ECG waveform could be beneficial for estimating left cardiac remodeling in dogs with MMVD.

This study aimed to evaluate whether each ECG parameter was useful for estimating left cardiac remodeling and investigate whether the rate of change in the ECG waveforms in the same individual reflected left cardiac remodeling in dogs with MMVD.

## Materials and Methods

### Ethical approval

The study protocol was approved by the Ethics Committee of the Veterinary Medical Teaching Hospital of Nippon Veterinary and Life Science University (Approval no. R3-3).

### Study period and location

This retrospective study included 160 client-owned dogs that were examined from January 2012 to December 2021 at Veterinary Medical Teaching Hospital of Nippon Veterinary and Life Science University.

### Dogs

Dogs with a normal heart or MMVD who had a medical history, and underwent physical examination, ECG, thoracic radiography, and echocardiography were included in the study. Data were collected from dogs that required cardiovascular examination or reassessment of MMVD. Among the dogs with a normal heart, we included those who had undergone cardiac examination as a second opinion when cardiac enlargement was suspected on thoracic radiography. In these cases, as no heart murmur was heard and there were no findings of cardiac enlargement in the imaging test and ECG, they were judged to have normal hearts. All pet owners provided informed consent before the inclusion of their dogs in the study. All examinations were performed in a quiet room without sedation. We excluded dogs with ECG waveforms that were difficult to assess because of arrhythmia (e.g., atrial fibrillation, ventricular tachycardia, and second- and third-degree atrioventricular block), and those with electrolyte imbalance or athletic heart syndrome, such as trained sledge dogs [7, 14]. Changes in the electrical axis caused by the right cardiac enlargement could affect the association between ECG waveforms and left cardiac remodeling. Hence, this study did not include dogs with post-capillary pulmonary hypertension secondary to left-sided MMVD. In this study, according to the ACVIM consensus statement, a tricuspid regurgitation jet velocity of ≥3.0 m/s and/or a pulmonary regurgitation jet velocity of ≥2.5 m/s, and anatomical abnormalities detected by echocardiography (e.g., pulmonary artery enlargement: pulmonary artery/Ao ratio >1.0, flattening of the interventricular septum [especially systolic flattening]) were diagnosed as post-capillary pulmonary hypertension [15]. We excluded dogs with diseases other than those mentioned above and those treated for conditions other than MMVD. However, dogs with benign skin tumors, mild oral disease, or mild patellar dislocation were judged to have no effect on the results and were thus included in this study.

**Figure-1:**
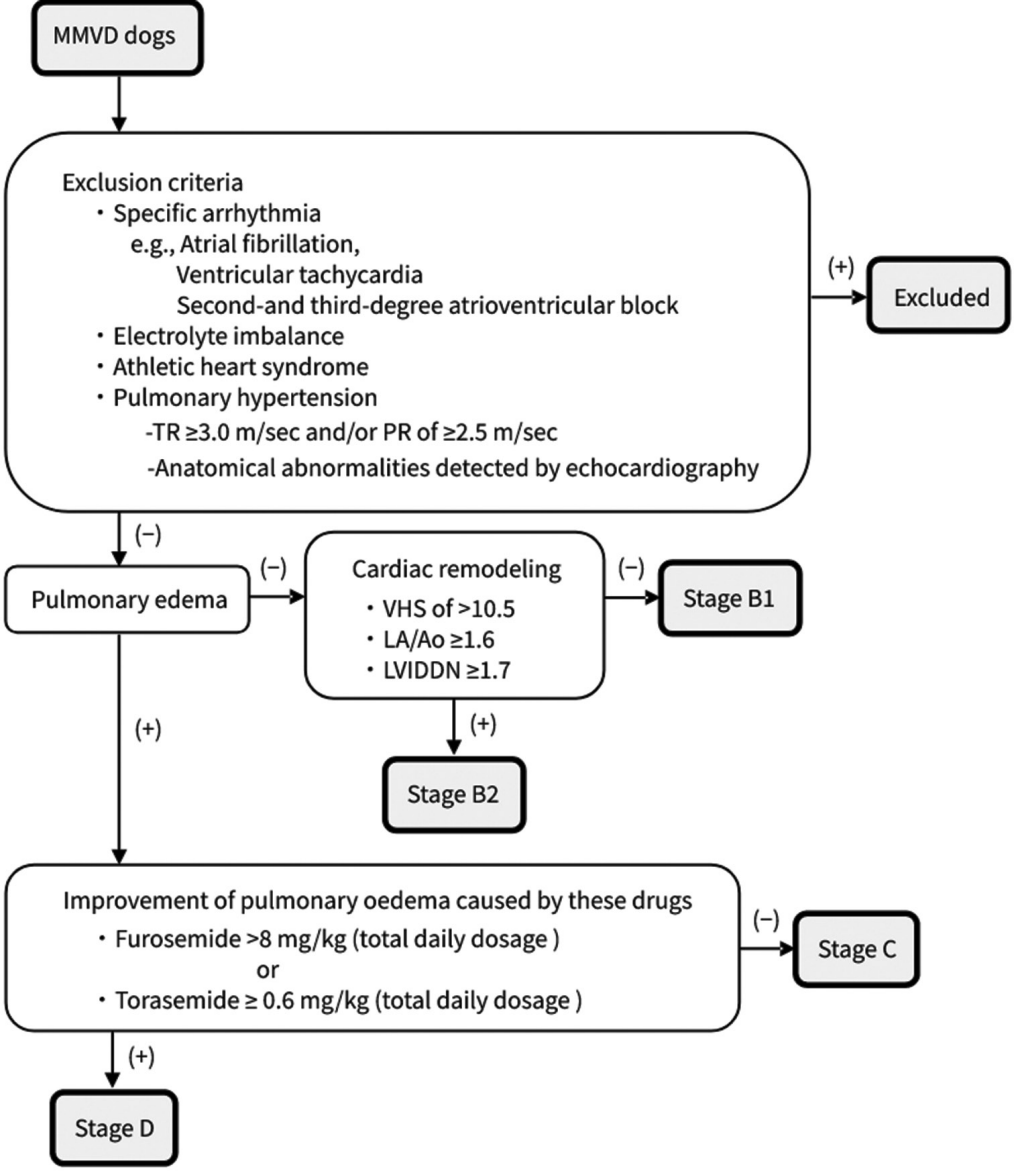
Flowchart for the selection and exclusion of dogs with MMVD in this study. MMVD=Myxomatous mitral valve disease, PR=Pulmonary regurgitation, TR=Tricuspid regurgitation, VHS=Vertebral heart size, LA/Ao=Left atrial/aortic ratio, LVIDDN=Left ventricular internal diameter in diastole normalized for body weight.

The dogs included in this study were classified according to the most recent consensus statement by ACVIM [3]. A flowchart of the selection and exclusion of dogs with MMVD in this study is shown in Figure-1. Stage A is characterized by a high risk of developing heart disease without any currently identifiable structural disorders in the heart. Among the dogs with MMVD without congestive heart failure, those with cardiac remodeling were considered to have stage B2 disease. The diagnostic criteria for left cardiac remodeling were as follows: radiographic VHS >10.5, echocardiographic LA/Ao in the right-sided short-axis view in early diastole ≥1.6, and LVIDDN (measured from the right parasternal short-axis view at the papillary muscle level) ≥1.7 [3]. Dogs with either current or previous clinical signs of heart failure caused by MMVD were classified into Stage C. Dogs with end-stage MMVD, in which the clinical signs of heart failure were refractory to standard treatment, were classified as Stage D. Stage D included dogs requiring furosemide at a total daily dosage of >8 mg/kg or torasemide with an equivalent dosage (≥0.6 mg/kg) administered concurrently with other medications at standard doses, which reportedly controlled clinical heart failure symptoms [3, 16]. Stages C and D were characterized by pulmonary edema confirmed via medical history and physical examination, thoracic radiography, and echocardiography. In this study, the control group included dogs with normal hearts and those with stage A. Meanwhile, dogs with Stages B1, B2, C, and D were included in the MMVD group. In addition, dogs in the control group and those with Stage B1 were classified into the non-left cardiac remodeling group. Dogs with Stages B2, C, and D were classified into the left cardiac remodeling group.

### Clinical variables

The clinical variables in the current study were age, body weight, heart rate, murmur intensity, VHS, VLAS, LA/Ao, LVIDDN, P-wave duration, PR interval, QRS complex duration, P-wave amplitude, R-wave amplitude, and mean electrical axis.

ECG was performed by holding the animal in the right recumbent position using limb leads. All ECG parameters were recorded using an ECG monitor (Cardisuny D700, Fukuda M-E Kogyo Co. Ltd., Japan). The ECG leads were attached to the skin using alligator clips at the plantar or caudal aspect of the left and right foreleg over or just distal to the olecranon, and at the cranial aspect of the left and right hind limbs over the patellar ligament. The electrode attachment points were moistened with 70% denatured alcohol, and the ECG was recorded at a frequency range of 0.05–100 Hz. We did not use a filter to minimize the baseline interference to prevent a decrease in the wave amplitudes. The ECG measurement conditions were 50 mm/s and 2.5, 5, and 10 mm/mV, respectively.

All ECG waveforms were measured using lead II, and the waveforms measured using lead I and lead III were used to calculate the average electrical axis. The measurements were performed by the first author using calipers. The baseline was from the end of the T wave to the next P-wave. The waveform amplitude was from the upper edge of the baseline to the top of the wave for positive waves, and from the lower edge of the baseline to the bottom of the wave for negative waves. The duration and interval between the inflection points of each waveform were measured. The measured value of each waveform was calculated by obtaining the average of the continuous waveforms of six beats in which the immediately preceding baseline was not pitched [10]. To measure the mean electric axis, the amplitudes of the waveforms of each QRS complex in inductions I and III were first measured. The sum of the measured values was subsequently plotted for induction I and III. Next, a perpendicular line was drawn from the points plotted on the two types of induction, and a line was drawn connecting the center point (the intersection points of inductions I and III) to the intersection point of the two points. Thereafter, we measured the angle between the anode of induction I and the straight line described above using the anode of induction I as a reference [7].

### Thoracic radiography

Right-lateral and dorsoventral radiographic images of the thorax were routinely obtained for each dog using a commercially available digital radiography system. Assessments of VHS and VLAS were done as previously described by Buchanan and Bücheler [17] and Malcolm *et al*. [18] using right-lateral radiographic images. The VHS was calculated as the sum of the long and short axes of the heart. The long axis was measured from the ventral border of the carina to the heart apex. The short-axis, which was perpendicular to the long axis, was defined as the line with the maximum dimension of the heart in the central-third region, including the right atrium and left heart chambers [17]. To measure VLAS, a line was drawn and measured from the ventral border of the carina to the dorsal border of the caudal vena cava, where it intersected the LA. The same line length was drawn beginning at the cranial edge of the fourth thoracic vertebrae and expressed in vertebral body units to the nearest 0.1 vertebrae as VLAS [18, 19].

### Echocardiography

Experienced echocardiographers performed echocardiography using the conventional method with an ultrasonographic unit fitted with a 6–12-MHz probe. ECG (lead II) was also performed during echocardiography. Each dog was manually restrained first on the right and subsequently on the left, while in the lateral recumbent position. The LA and Ao dimensions were obtained from the right parasternal short-axis view in early diastole, and the LA/Ao was calculated [20]. The Ao dimension was measured by placing the first caliper on the midpoint of the convex curvature of the right coronary Ao sinus wall, and the second caliper at the point where the Ao wall and non-coronary and left coronary Ao cusps merged. From this point, the LA dimension was measured by extending the Ao line to the blood-tissue interface of the LA wall. The LVIDDN was measured from the right parasternal short-axis view at the papillary muscle level using M-mode echocardiography, whereas LVIDDN was calculated using the following formula [3]:

LVIDDN = LVIDD [cm]/body weight [kg]^0.294^.

### Rate of change in ECG waveforms

Among the dogs with examination data that could be obtained multiple times during the study period, those that did not progress to Stage B2 were classified under the non-progressive group. Those that progressed from Stages B1 to B2 were classified into progressive groups. In the non-progressive group, the first data were obtained at the first visit (PRE) and the last data were obtained during the study period (POST), whereas in the progressive group, the first data were obtained at the first visit (PRE) and the last data when stage B2 had progressed (POST). These data were used to calculate the rate of change of the ECG waveforms using the following formula:


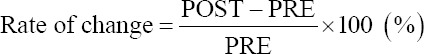


### Statistical analysis

Statistical analyses were performed using SPSS Statistics (version 24.0; IBM Corp., NY, USA). The normality of the data was assessed using the Shapiro–Wilk test. Continuous data are presented as median (interquartile range). In this study, the heart murmur intensity was considered a continuous variable. The Kruskal–Wallis test was used to compare the ACVIM stages and ECG parameters. If a significant difference was observed, all pairs of medians were compared using the Steel–Dwass test. In addition, Stages C and D were combined into one group because of the small number of cases.

The association between ECG parameters and each clinical variable was determined using the Spearman’s rank correlation coefficient. Next, to assess each imaging test parameter as an independent predictor, ECG parameters that showed statistically significant correlations with the imaging test parameters (p < 0.05) by Spearman’s rank correlation coefficient were included in the multiple linear regression analysis using the forward-backward stepwise selection technique. A standard partial regression coefficient more than 0.2 was judged to be significantly related. In addition, residues had a normal distribution when assessed using the Shapiro–Wilk test, and the assumptions of the model were assessed and satisfied.

Receiver operating characteristic (ROC) curve analysis was performed to calculate the area under the ROC curve (AUC), and the sensitivity and specificity of the ECG parameters for their ability to discriminate between the two left cardiac remodeling groups (non-left cardiac remodeling group and left cardiac remodeling group). We also compared the AUC indicated by each ECG parameter with the AUC versus the area under the diagonal line, using a corresponding t-test. ECG parameters for which no significant difference was found between the AUC and the diagonal line using a corresponding t-test were judged as unable to discriminate the left heart remodeling. The cutoff value was obtained from the point closest to the top left of the ROC curve, with sensitivity on the vertical axis and 1-specificity on the horizontal axis. We also investigated the discriminatory ability of Stages B1 and B2, excluding the control group, and Stages C and D.

To evaluate intra-individual variations in the ECG parameters, the measured values of PRE and POST were compared using the Wilcoxon signed-rank test. Furthermore, the rate of change of each ECG parameter ([POST–PRE]/PRE × 100) was calculated to eliminate the inter-individual variability and compared between the non-progressive and progressive groups using the Wilcoxon signed-rank test. The rate of change of each ECG parameter with significant differences between the non-progressive and progressive groups was evaluated to assess the discriminatory ability for the left cardiac remodeling using ROC curve analyses; p < 0.05 were considered statistically significant.

## Results

### Dogs

In total, the median age and weight of the dogs in the control group (n = 20) were 5.3 (interquartile range: 1.4–8.3) years and 5.40 (2.83–7.02) kg, respectively. Meanwhile, the median age and weight of the dogs in the MMVD group (n = 140; ACVIM classification: B1, n = 87; B2, n = 44; C, n = 3; D, n = 6) were 11.5 (interquartile range: 8.9–12.9) years and 4.60 (3.25–7.08) kg, respectively (Table-1). The breed distributions in the control and MMVD groups are shown in Table-2.

**Table-1:** Characteristics of dogs in the control and MMVD groups.

Characteristics	Control group	MMVD group			

		B1	B2	C	D
Number (head)	20	87	44	3	6
Sex (head)					
Male	7	20	11	1	3
Female	5	30	12	0	0
Neuterd male	5	22	16	1	1
Neuterd female	3	15	5	1	2
Age (years)	5.3	11.5^a^	11.8^a^	9.6	11.2^a^
	(1.4–8.3)	(8.4–12.9)	(9.4–13.1)	(8.9–12.0)	(8.5–12.5)
Body weight (kg)	5.40	4.64	4.50	14.20	3.30
	(2.83–7.02)	(3.35–6.75)	(3.13–7.45)	(6.85–14.30)	(3.03–6.52)
Heart rate (bpm)	120	120	136	156	138
	(100–144)	(108–126)	(120–150)	(130–168)	(119–157)
Murmur (Levine)	–	3	5^b^	5	5^b^
		(1–6)	(3–5)	(3–6)	(4–6)
VHS (v)	10.2	10.5	11.9^a,b^	12.5^a^	13.2^a,b^
	(8.4–11.2)	(8.7–12.9)	(10.6–15.6)	(11.6–15.2)	(11.6–13.7)
VLAS (v)	1.8	2.2^a^	2.6^a,b^	3.3	3.0^a,b^
	(1.2–2.5)	(1.1–2.9)	(1.5–4.1)	(2.9–3.6)	(3.0–3.3)
LA/Ao	1.31	1.51^a^	2.51^a,b^	3.08^a^	2.90
	(0.79–1.62)	(0.92–2.87)	(1.64–3.22)	(1.83–3.16)	(1.61–3.54)
LVIDDN	1.57	1.65	2.14^a,b^	2.05	2.55^a,b^
	(1.21–2.04)	(1.55–2.43)	(1.77–2.24)	(1.94–2.63)	(2.30–2.79)
P-wave duration (sec)	0.046	0.048	0.052^a,b^	0.056	0.055
	(0.039–0.048)	(0.044–0.053)	(0.049–0.055)	(0.040–0.068)	(0.046–0.056)
PR interval (sec)	0.086	0.088	0.094	0.095	0.092
	(0.076–0.110)	(0.080–0.098)	(0.087–0.104)	(0.071–0.100)	z(0.077–0.120)
QRS complex duration (sec)	0.032	0.043^a^	0.051^a,b^	0.047	0.055^a^
	(0.028–0.036)	(0.035–0.052)	(0.045–0.058)	(0.040–0.072)	(0.037–0.061)
P-wave amplitude (mV)	0.179	0.213	0.216	0.213	0.226
	(0.148–0.230)	(0.152–0.304)	(0.159–0.302)	(0.162–0.306)	(0.197–0.253)
R-wave amplitude (mV)	1.97	2.29	2.68	3.08	2.21
	(1.59–2.64)	(1.78–2.90)	(1.91–3.19)	(2.32–3.65)	(1.68–2.95)
Mean electrical axis (°)	72.8	69.9	70.6	77.5	82.5
	(60.7–83.9)	(55.4–79.2)	(49.9–85.8)	(49.7–82.4)	(74.8–85.2)

Data are presented as median (interquartile range),

a Significantly different from the control group (p < 0.05),

b Significantly different from stage B1 (p < 0.05),

^c^Significantly different from stage B2 (p < 0.05). MMVD=Myxomatous mitral valve disease, VHS=Vertebral heart size, VLAS=Vertebral left atrial size, LA/Ao=Left atrial/aortic ratio, LVIDDN=Left ventricular internal diameter in diastole normalized for body weight.

**Table-2:** Breed distribution in the control and MMVD groups.

Breed	Control group	MMVD group
Beagle	0	2
Cavalier King Charles Spaniel	1	19
Chihuahua	2	37
Dachshund	2	8
Maltese	1	15
Miniature Schnauzer	0	5
Papillon	1	4
Pomeranian	1	5
Poodle (Toy)	2	11
Shih Tzu	0	12
Yorkshire Terrier	1	2
Others	9	20

MMVD=Myxomatous mitral valve disease.

### Comparison of ECG waveforms between each ACVIM stage

**Figure-2:**
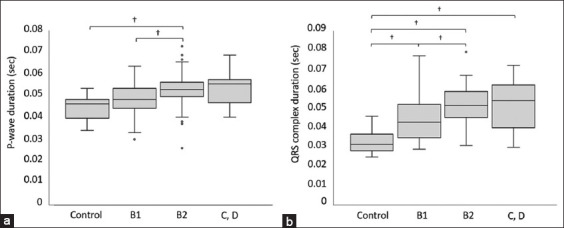
Comparison of electrocardiogram parameters between each American College of Veterinary Internal Medicine stage group. (a) P-wave duration, (b) QRS complex duration. The box represents the interquartile range and the line within the median. The whiskers reflect the most extreme values with an interquartile range of <1.5, which is beyond the upper or lower quartiles. ○, outlier; †, p < 0.01.

A significant difference was observed between the P-wave and QRS complex durations when each ECG waveform between the control and ACVIM stage groups was compared (Figure-2). There was a significant difference in the P-wave duration between the Stage B2 group and the control or Stage B1 group (p < 0.01 for all). The QRS complex duration was significantly different between the B1, B2, C, and D groups and the control group (p < 0.01 for all), and between the B1 and B2 groups (p < 0.01). No significant differences were found between the different stages in terms of the PR interval, P-wave amplitude, R-wave amplitude, and mean electrical axis.

### Correlation between each clinical variable

The results of the univariate analysis are presented in Table-3. In the multiple regression analysis, including statistically significant ECG parameters, VHS and VLAS were significantly associated with the duration of the P-wave and QRS complex. In addition, LA/Ao was only associated with the P-wave duration and LVIDDN was only associated with the QRS complex duration (Table-4).

**Table-3:** Correlation coefficient of electrocardiogram parameters and clinical variables based on a correlation analysis.

Variables	Age (years)		Body wight (kg)		Heart rate (bpm)		VHS (v)	
			
	Correlation coefficient	p-value	Correlation coefficient	p-value	Correlation coefficient	p-value	Correlation coefficient	p-value
P-wave duration (sec)	0.11	0.18	0.22	<0.01	0.02	0.80	0.24	<0.01
PR interval (sec)	0.01	0.90	0.45	<0.01	0.11	0.16	0.14	0.05
QRS complex duration (sec)	0.23	<0.01	0.19	<0.05	0.02	0.79	0.34	<0.01
P-wave amplitude (mV)	0.01	0.92	0.20	<0.05	0.18	0.83	0.01	0.88
R-wave amplitude (mV)	0.09	0.27	0.04	0.61	0.01	0.84	0.16	<0.05
Mean electrical axis (°)	0.01	0.89	0.04	0.66	0.07	0.41	0.03	0.68

	VLAS (v)		LA/Ao		LVIDDN			
		
	Correlation coefficient	p-value	Correlation coefficient	p-value	Correlation coefficient	p-value

P-wave duration (sec)	0.35	<0.01	0.37	<0.01	0.28	<0.01		
PR interval (sec)	0.06	0.43	0.12	0.15	0.15	0.06		
QRS complex duration (sec)	0.39	<0.01	0.39	<0.01	0.39	<0.01		
P-wave amplitude (mV)	0.25	<0.01	0.10	0.21	0.05	0.56		
R-wave amplitude (mV)	0.15	0.08	0.19	<0.05	0.16	<0.05		
Mean electrical axis (°)	0.03	0.72	0.04	0.63	0.03	0.73		

VHS=Vertebral heart size, VLAS=Vertebral left atrial size, LA/Ao=Left atrial/aortic ratio, LVIDDN=Left ventricular internal diameter in diastole normalized for body weight.

**Table-4:** Multiple regression analysis performed using parameters with significant differences in the univariate analysis.

Dependent variable	Independent variable	Partial regression coefficient (95% CI)	Standard partial regression coefficient	p-value
VHS (v)	P-wave duration (sec)	39.71	0.205	<0.05
		(9.66–69.76)		
	QRS complex duration (sec)	32.71	0.273	<0.01
		(14.48–50.95)		
VLAS (v)	P-wave duration (sec)	13.21	0.257	<0.01
		(8.61–34.49)		
	QRS complex duration (sec)	13.21	0.262	<0.01
		(5.42–21.00)		
LA/Ao	P-wave duration (sec)	37.20	0.314	<0.01
		(19.20–55.19)		
	QRS complex duration (sec)	13.80	0.196	<0.05
		(2.75–24.84)		
LVIDDN	P-wave duration (sec)	10.58	0.199	<0.05
		(2.14–19.02)		
	QRS complex duration (sec)	9.18	0.283	<0.01
		(4.14–14.23)		

CI=Confidence interval, VHS=Vertebral heart size, VLAS=Vertebral left atrial size, LA/Ao=Left atrial/aorta ratio, LVIDDN=Left ventricular internal diameter in diastole normalized for body weight.

### Discriminatory ability of ECG waveform for the left cardiac remodeling

**Figure-3:**
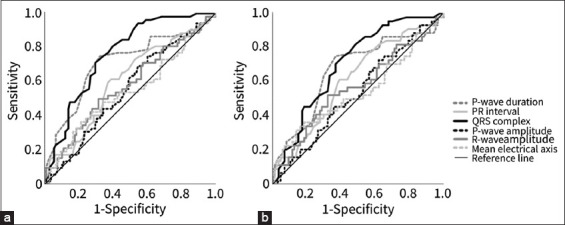
Comparison of the AUC of electrocardiogram parameters for the left cardiac remodeling through ROC curve analysis. (a) The ROC curve analyses using the non-left cardiac remodeling group and the left cardiac remodeling group, (b) the ROC curve analyses using dogs with Stages B1 and B2. ROC, receiver operating characteristic; AUC, area under the receiver operating characteristic curve.

In the ROC curve analyses using the non-left cardiac remodeling group and the left cardiac remodeling group, the AUC of the ROC curve was significantly larger than the area under the diagonal line for P-wave duration, PR interval, and QRS complex duration (Figure-3a). However, there were no significant differences in the AUC concerning the P-wave amplitude, R-wave amplitude, and average electrical axis compared with the area under the diagonal line. The sensitivity and specificity of each ECG waveform are listed in Table-5.

**Table-5:** Discriminatory ability of the left cardiac remodeling of electrocardiogram wave forms (non-left cardiac remodeling group and the left cardiac remodeling group).

Electrocardiography parameters	Cut-off value	AUC 95% CI	Sensitivity (%) 95% CI	Specificity (%) 95% CI	PPV (%) 95% CI	NPV (%) 95% CI	Likelihood ratio 95% CI
P-wave duration	0.050 sec	0.70	75.0	67.6	52.7	84.9	2.31
		0.61–0.80	0.61–0.84	0.58–0.75	0.41–0.63	0.75–0.90	1.69–3.16
PR interval	0.093 sec	0.60	61.5	61.1	43.2	76.7	1.85
		0.50–0.69	0.48–0.73	0.51–0.69	0.32–0.54	0.66–0.84	1.16–2.95
QRS complex duration	0.047 sec	0.76	71.2	70.4	53.6	83.5	2.40
		0.68–0.83	0.57–0.81	0.61–0.78	0.42–0.64	0.76–0.89	1.71–3.36
P-wave amplitude	0.20 mV	0.55	61.5	50.9	37.6	73.3	1.25
		0.46–0.64	0.48–0.73	0.41–0.60	0.28–0.48	0.62–0.82	0.94–1.67
R-wave amplitude	2.67 mV	0.58	50.0	68.5	43.3	74.0	1.58
		0.48–0.67	0.36–0.63	0.59–0.76	0.31–0.55	0.64–0.81	1.07–2.34
Average electrical axis	77.54°	0.54	48.1	65.7	40.3	72.4	1.40
		0.44–0.64	0.35–0.61	0.56–0.74	0.29–0.52	0.62–0.80	0.95–2.06

AUC=Area under the receiver operating characteristic curve, CI=Confidence interval, PPV=Positive predictive value, NPV=Negative predictive value.

The results in the ROC curve analyses using stage B1 and B2 dogs were similar to those using the non-left and left cardiac remodeling groups. The AUC of the ROC curve was significantly larger than the area under the diagonal line for P-wave duration, PR interval, and QRS complex duration (Figure-3b). The sensitivity and specificity of each ECG waveform are listed in Table-6.

**Table-6:** Discriminatory ability of the left cardiac remodeling of electrocardiogram wave forms (ACVIM Stages B1 and B2).

Electrocardiography parameters	Cut-off value	AUC 95% CI	Sensitivity (%) 95% CI	Specificity (%) 95% CI	PPV (%) 95% CI	NPV (%) 95% CI	Likelihood ratio 95% CI
P–wave duration	0.050 sec	0.67	75.0	63.2	50.8	83.3	2.03
		0.56–0.77	0.60–0.85	0.52–0.72	0.38–0.62	0.72–0.90	1.47–2.81
PR interval	0.093 sec	0.61	61.4	60.9	44.3	75.7	1.57
		0.51–0.71	0.46–0.74	0.50–0.70	0.32–0.56	0.64–0.84	1.10–2.23
QRS complex duration	0.047 sec	0.70	70.0	63.2	49.2	80.9	1.91
		0.61–0.79	0.55–0.81	0.52–0.72	0.37–0.61	0.70–0.88	1.37–2.67
P–wave amplitude	0.23 mV	0.53	45.5	64.4	39.2	70.0	1.27
		0.43–0.64	0.31–0.59	0.53–0.73	0.27–0.52	0.59–0.78	0.83–1.96
R–wave amplitude	2.67 mV	0.56	52.6	67.8	45.1	73.8	1.62
		0.46–0.67	0.37–0.66	0.57–0.76	0.32–0.58	0.63–0.82	1.07–2.46
Average electrical axis	72.80°	0.54	47.7	58.6	36.8	68.9	1.15
		0.43–0.65	0.33–0.62	0.48–0.68	0.25–0.49	0.57–0.78	0.77–1.71

AUC=Area under the receiver operating characteristic curve, CI=Confidence interval, PPV=Positive predictive value, NPV=Negative predictive value.

### Rate of change in ECG waveforms

**Figure-4:**
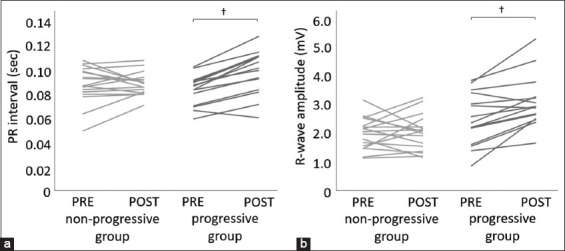
Comparison of PRE and POST electrocardiogram waveforms between the non-progressive and progressive groups. (a) PR interval, (b) R-wave amplitude. †, p < 0.01. Non-progressive group, dogs that did not progress to stage B1; progressive groups, dogs that progressed from Stage B1 to B2; PRE, initial data; POST, last data.

**Figure-5:**
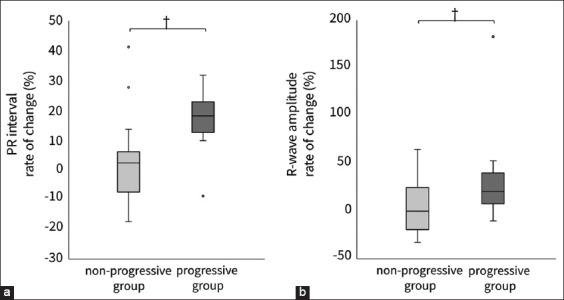
Comparison of the rate of change calculated using PRE and POST electrocardiogram waveforms. (a) PR interval, (b) R-wave amplitude. The box represents the interquartile range and the line within the median. The whiskers reflect the most extreme values with an interquartile range of <1.5, which is greater than the upper or lower quartiles. ○, outlier; †, p < 0.01. Non-progressive group, dogs that did not progress to Stage B1; progressive group, dogs that progressed from Stage B1 to B2.

**Figure-6:**
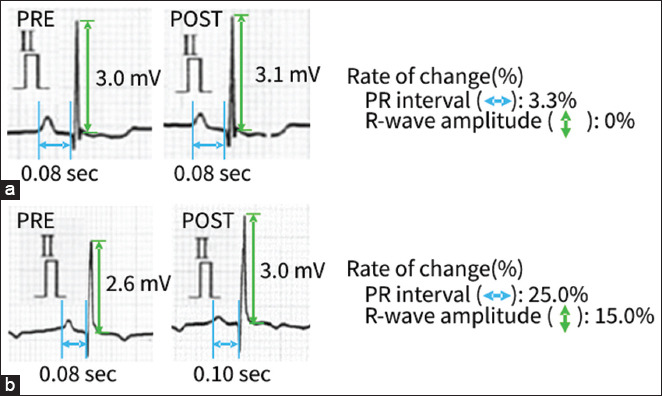
(a and b) Representative tracings of electrocardiogram waveforms for the non-progressive group and the progressive group. Non-progressive group, dogs that did not progress to stage B1; progressive group, dogs that progressed from Stage B1 to B2; PRE, initial data; POST, last data.

The PRE and POST ECG waveforms of the non-progressive (n = 17) and progressive (n = 15) groups were compared. The median period from PRE to POST was 0.9 (interquartile range: 0.6–1.3) years in the non-progressive group and 0.6 (0.5–1.2) years in the progressive group. Figure-4 shows the results of the comparison with the actual measurements. There were no significant differences in the PRE and POST ECG parameters in the non-progressive group. However, significant differences were observed between the PRE and POST PR intervals and R-wave amplitudes in the progressive group (Figure-4; both p < 0.01). Furthermore, the rate of change in both PR interval and R-wave amplitude in the progressive group was significantly higher than that in the non-progressive group (Figure-5; both p < 0.01). Representative ECG tracings of the non-progressive and progressive groups are shown in Figure-6.

**Figure-7:**
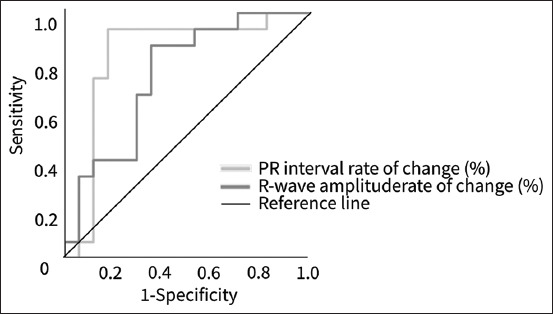
Comparison of the AUC of the rate of change calculated using the electrocardiogram waveforms for the left cardiac remodeling through ROC curve analysis. ROC=Receiver operating characteristic, AUC=Area under the receiver operating characteristic curve.

The AUC of the ROC curve was significantly larger than the area under the diagonal line for the rate of change in the PR interval and R-wave amplitude (Figure-7). The rate of change of the ECG waveforms is shown in Table-7.

**Table-7:** Discriminatory ability of left cardiac remodeling of rate of change in PR interval and R-wave amplitude.

Electrocardiography parameters	Cut-off value	AUC 95% CI	Sensitivity (%) 95% CI	Specificity (%) 95% CI	PPV (%) 95% CI	NPV (%) 95% CI	Likelihood ratio 95% CI
PR interval rate of change	9.7%	0.82	86.7	82.4	81.3	87.5	4.92
		0.65–0.99	0.62–0.96	0.59–0.93	0.57–0.93	0.64–0.96	1.72–13.97
R wave amplitude rate of change	6.7%	0.74	86.7	64.7	68.4	84.6	2.45
		0.57–0.92	0.62–0.96	0.41–0.82	0.46–0.84	0.57–0.95	1.07–2.34

AUC=Area under the receiver operating characteristic curve, CI=Confidence interval, PPV=Positive predictive value, NPV=Negative predictive value.

## Discussion

In this study, although the P-wave and QRS complex durations were found to be significantly associated with imaging test parameters for the left cardiac remodeling, they did not have sufficient discriminative accuracy for estimating left cardiac remodeling. However, the rate of change in the PR interval and R-wave amplitude over time improved the diagnostic ability for left cardiac remodeling. Although several studies have evaluated ECGs in dogs with MMVD in recent years [21, 22], to the best of our knowledge, this is the first study to focus on individual differences in ECG waveforms and show that the assessment of changes in ECG waveforms over time may be useful for estimating left cardiac remodeling in dogs with MMVD.

In this study, only the QRS complex duration showed a significant difference between the control and Stage B1 groups, and this waveform was associated with the imaging test parameters in the multiple regression analysis. This may reflect left cardiac remodeling that does not extend to Stage B2. The duration of the QRS complex reflects ventricular depolarization [7]. Only VLAS and LA/Ao showed significant differences between the control and Stage B1 groups, whereas VHS and LVIDDN did not show any difference. However, given that a small number of dogs that deviated from the VHS and LVIDDN criteria were included in Stage B1, QRS complex duration may reflect earlier LV enlargement.

In contrast to the previous research, the PR interval, P-wave amplitude, R-wave amplitude, and mean electrical axis did not correlate with any of the imaging test parameters and showed a low discriminatory ability against left cardiac remodeling [7]. There are several possible explanations for this finding. As the waveforms of six consecutive beats were averaged and calculated, the P-wave may have been affected by a wandering pacemaker. Wandering pacemakers are often observed in brachycephalic dog breeds and older dogs [23]. In this study, most of the dogs were short-headed and older, including the Chihuahuas and Cavalier King Charles Spaniels. In this study, as in the previous reports, the PR interval was positively correlated with body weight [24]. This association might have masked the association between PR interval and left cardiac remodeling. When the thorax is deeper in the R-wave amplitude of dogs, the waveform amplitude is larger [7, 25]. In dogs with a narrow chest, the distance from the heart to the chest wall is short. As for the average electric axis, the orientation of the heart in the thoracic cavity differs due to variations in thoracic morphology based on breed [7]. Based on the above-mentioned data, individual differences such as body weight and chest circumference may explain why these ECG parameters did not reflect the pathological condition of MMVD in this study.

As mentioned above, the accuracy of reference values in identifying the left cardiac enlargement without considering the effects of body weight and physique (particularly thoracic morphology) remains unclear. Based on this finding, we investigated whether the rate of change in ECG waveforms in the same individual differed significantly between the non-progressive and progressive groups. The results showed that only the PR interval and R-wave amplitude significantly changed with MMVD progression in the same individual. The PR interval reflects the time taken for the depolarizing wave to be conducted through the atria, atrioventricular node, and the bundle [23]. A prolonged stimulus conduction time has been reported to comprehensively reflect the estimation of both advanced structural and electrical remodeling [26]. As the PR interval is determined by the conduction time from the sinus node to the ventricle, it integrates information about many parts of the conduction system of the heart [27]. Therefore, the rate of change in the PR interval may have performed better than the durations of the P-wave and the QRS complex, which only reflects LA or LV remodeling. The R-wave may have been enhanced by the shortening of the distance between the R-wave and chest wall owing to the axial deviation caused by cardiac enlargement [7]. However, because the R-wave amplitude varies among individuals due to differences in contours of the thorax or amounts of adipose tissue, only the rate of variation may have reflected left cardiac remodeling. The conduction time may be why the rate of change in the R-wave amplitude was better than that in the QRS complex duration. The QRS complex duration extends with an increase in intraventricular pressure and persistence of cardiac enlargement, as the resting potential of ventricular myocytes becomes shallower and the action potential amplitude decreases [28]. Therefore, it is possible that the prolongation of the QRS complex duration appears later than the increase in the R-wave amplitude associated with the left cardiac remodeling. It has been reported that only the P-wave and QRS complex durations reflect cardiac enlargement in dogs [29]. However, our results suggest that the PR interval and R-wave amplitude reflect left cardiac remodeling as much as or more than the P-wave and QRS complex durations if the effects of inter-individual variability are excluded from the study. Therefore, conducting regular ECGs in clinical settings and examining these items may facilitate decision-making regarding further imaging tests.

In this study, the heart rate did not significantly differ among the ACVIM stage groups. In dogs with MMVD, vagal nerve activity changes to sympathetic nerve activity with clinical stage progression [30]. The heart rate was measured as the instantaneous heart rate in the hospital. This was one reason for the lack of significant differences in heart rate in this study. There is a diurnal variation in the heart rate of dogs, which tends to increase in hospitals [31]. The resting heart rate measured at home may differ significantly between ACVIM stages. A previous study showed that the PR interval decreased with increasing heart rate in dogs with congestive heart failure [32]. Nevertheless, this finding should be further investigated because of the small number of dogs in Stages C and D in this study.

This study had some limitations. First, the number of dogs in Groups C and D was relatively low. This was because a few dogs with Stages C and D presented with dyspnea. Thus, ECG was considered inappropriate for these dogs, and they were not included in the study. Increasing the number of dogs with Stages C and D may improve the discriminatory ability of ECG waveforms for the left cardiac remodeling. Second, chest computed tomography (CT) scan was not performed in this study. Therefore, the extent to which the morphology of the thorax differed among the cases could not be determined. Further studies using CT scans should be conducted to determine the effect of different thoracic morphologies on each ECG waveform.

## Conclusion

The P-wave and QRS complex durations were significantly correlated with the left cardiac remodeling indicators in dogs with MMVD. However, the fluctuation rate of the PR interval and the rate of change in the R-wave amplitude in the same individual could be indicators of left cardiac remodeling. We believe that collecting data from more cases in the future and clarifying the relationship between body size differences, such as the thorax and ECG waveforms, will further confirm the significance of assessing intra-individual variations in ECG waveforms in dogs with MMVD.

## Authors’ Contributions

MO: Conception and design of the study, acquisition, analysis, interpretation of data, and drafting of the article. HO: Acquisition, analysis, and interpretation of data. HM: Review of the article. HH: Review of the article. YM: Review of the article. NT: Review of the article. All authors have read and approved the final manuscript.
